# Supplementary material for: Machine Learning Accelerates Crystallization for Structure Determination
Source: Angew Chem Int Ed Engl. 2026 May 4;65(25):e1218503. doi: 10.1002/anie.1218503 (PMC13266958; doi:10.1002/anie.1218503)

# checkCIF/PLATON report

Structure factors have been supplied for datablock(s) lcz027

THIS REPORT IS FOR GUIDANCE ONLY. IF USED AS PART OF A REVIEW PROCEDURE FOR PUBLICATION, IT SHOULD NOT REPLACE THE EXPERTISE OF AN EXPERIENCED CRYSTALLOGRAPHIC REFEREE.

No syntax errors found.  CIF dictionary  Interpreting this report

# Datablock: lcz027

---

| | | | |
|---|---|---|---|
| Bond precision: | C-C = 0.0247 A | | Wavelength=1.54184 |
| Cell: | a=23.3011(4) | b=9.4996(2) | c=15.8191(3) |
| | alpha=90 | beta=108.666(2) | gamma=90 |
| Temperature: | 100 K | | |

| | Calculated | Reported |
|---|---|---|
| Volume | 3317.39(12) | 3317.39(12) |
| Space group | P 21/c | P 1 21/c 1 |
| Hall group | -P 2ybc | -P 2ybc |
| Moiety formula | C26 H15 Ag3 F18 N6 O2 [+ solvent] | C15 H3 Ag3 F18 N6, C11 H12 O2 |
| Sum formula | C26 H15 Ag3 F18 N6 O2 [+ solvent] | C26 H15 Ag3 F18 N6 O2 |
| Mr | 1109.05 | 1109.05 |
| Dx,g cm-3 | 2.221 | 2.221 |
| Z | 4 | 4 |
| Mu (mm-1) | 15.380 | 15.380 |
| F000 | 2128.0 | 2128.0 |
| F000’ | 2138.30 | |
| h,k,lmax | 29,11,19 | 29,11,19 |
| Nref | 6788 | 6408 |
| Tmin,Tmax | 0.149,0.158 | 0.223,1.000 |
| Tmin’ | 0.066 | |

Correction method= # Reported T Limits: Tmin=0.223 Tmax=1.000
AbsCorr = MULTI-SCAN

Data completeness= 0.944 Theta(max)= 74.601

R(reflections)= 0.1154( 5426) wR2(reflections)= 0.2963( 6408)

S = 1.242 Npar= 479

---

The following ALERTS were generated. Each ALERT has the format
**test-name_ALERT_alert-type_alert-level**.
Click on the hyperlinks for more details of the test.

---

### Alert level A

PLAT971_ALERT_2_A Check Calcd Resid. Dens. 0.83Ang From Ag02 5.51 eA-3

**Author Response: This Alert is due to presence of residual density in the presence of heavy metal atom (Ag).**

PLAT971_ALERT_2_A Check Calcd Resid. Dens. 0.85Ang From Ag02 4.91 eA-3

**Author Response: This Alert is due to presence of residual density in the presence of heavy metal atom (Ag).**

PLAT971_ALERT_2_A Check Calcd Resid. Dens. 0.89Ang From Ag01 4.53 eA-3

**Author Response: This Alert is due to presence of residual density in the presence of heavy metal atom (Ag).**

PLAT971_ALERT_2_A Check Calcd Resid. Dens. 0.93Ang From Ag01 4.51 eA-3

**Author Response: This Alert is due to presence of residual density in the presence of heavy metal atom (Ag).**

---

### Alert level B

PLAT342_ALERT_3_B Low Bond Precision on C-C Bonds ............... 0.02474 Ang.

**Author Response: This Alert is due to presence of residual density in the presence of heavy metal atom (Ag).**

```
PLAT971_ALERT_2_B Check Calcd Resid. Dens.  1.11Ang From O00X           3.12 eA-3
```

**Author Response: This Alert is due to presence of residual density in the presence of heavy metal atom (Ag).**

```
PLAT971_ALERT_2_B Check Calcd Resid. Dens.  0.87Ang From C01I           2.74 eA-3
```

**Author Response: This Alert is due to presence of residual density in the presence of heavy metal atom (Ag).**

```
PLAT971_ALERT_2_B Check Calcd Resid. Dens.  0.88Ang From F5             2.74 eA-3
```

**Author Response: This Alert is due to presence of residual density in the presence of heavy metal atom (Ag).**

```
PLAT972_ALERT_2_B Check Calcd Resid. Dens.  0.89Ang From Ag02          -2.62 eA-3
```

**Author Response: This Alert is due to presence of residual density in the presence of heavy metal atom (Ag).**

---

**Alert level C**

```
DIFMX02_ALERT_1_C  The maximum difference density is > 0.1*ZMAX*0.75
            The relevant atom site should be identified.
PLAT082_ALERT_2_C High R1 Value ..................................       0.12 Report
PLAT084_ALERT_3_C High wR2 Value (i.e. > 0.25) ...................       0.30 Report
PLAT094_ALERT_2_C Ratio of Maximum / Minimum Residual Density ....       2.04 Report
PLAT097_ALERT_2_C Large Reported Max.  (Positive) Residual Density       4.60 eA-3
PLAT213_ALERT_2_C Atom F00K            has ADP max/min Ratio .....        3.2 prolat
PLAT220_ALERT_2_C NonSolvent   Resd 1  F   Ueq(max)/Ueq(min) Range        3.1 Ratio
PLAT234_ALERT_4_C Large Hirshfeld Difference F00N     --C010     .       0.17 Ang.
PLAT362_ALERT_2_C Short  C(sp3)-C(sp2) Bond  C018     - C00W     .       1.40 Ang.
PLAT362_ALERT_2_C Short  C(sp3)-C(sp2) Bond  C00R     - C010     .       1.41 Ang.
PLAT906_ALERT_3_C Large K Value in the Analysis of Variance ......     14.550 Check
PLAT906_ALERT_3_C Large K Value in the Analysis of Variance ......      4.869 Check
PLAT906_ALERT_3_C Large K Value in the Analysis of Variance ......      2.001 Check
PLAT911_ALERT_3_C Missing FCF Refl Between Thmin & STh/L=    0.600         79 Report
               22  0  0,   0  4  2,  25  1  2,   1 10  3,  19  6  3,  -6 11  4,
                4  0  4,  16  8  4,  21  4  4,  22  4  4,  23  0  4, -12 10  5,
               15  8  5,  16  7  5,  18  4  5,  20  4  5, -23  6  6, -17  4  6,
              -25  5  7, -19  4  7,  19  4  7, -23  3  8, -23  4  8, -22  3  8,
               -9 10  8,  -4  9  8,  -3  9  8,   4  0  8,   9  8  8, -25  2  9,
                   (   49 More Missing: see the .ckf listing file)
PLAT934_ALERT_3_C Number of (Iobs-Icalc)/Sigma(W) > 10 Outliers ..          1 Check
                9  1 14,
PLAT971_ALERT_2_C Check Calcd Resid. Dens.  0.91Ang From Ag02           2.44 eA-3
```

**Author Response: This Alert is due to presence of residual density in the presence of heavy metal atom (Ag).**

```
PLAT971_ALERT_2_C Check Calcd Resid. Dens.  1.00Ang From Ag02           2.24 eA-3
```

**Author Response: This Alert is due to presence of residual density in the presence of heavy metal atom (Ag).**

```
PLAT971_ALERT_2_C Check Calcd Resid. Dens.  1.00Ang From F2             2.22 eA-3
```

**Author Response: This Alert is due to presence of residual density in the presence of heavy metal atom (Ag).**

```
PLAT971_ALERT_2_C Check Calcd Resid. Dens.  0.83Ang From Ag01           2.15 eA-3
```

**Author Response: This Alert is due to presence of residual density in the presence of heavy metal atom (Ag).**

```
PLAT971_ALERT_2_C Check Calcd Resid. Dens.  0.85Ang From Ag03           2.15 eA-3
```

**Author Response: This Alert is due to presence of residual density in the presence of heavy metal atom (Ag).**

```
PLAT971_ALERT_2_C Check Calcd Resid. Dens.  0.59Ang From C013           2.14 eA-3
```

**Author Response: This Alert is due to presence of residual density in the presence of heavy metal atom (Ag).**

```
PLAT971_ALERT_2_C Check Calcd Resid. Dens.  0.27Ang From C018           2.06 eA-3
```

**Author Response: This Alert is due to presence of residual density in the presence of heavy metal atom (Ag).**

```
PLAT971_ALERT_2_C Check Calcd Resid. Dens.  1.15Ang From Ag03           2.03 eA-3
```

**Author Response: This Alert is due to presence of residual density in the presence of heavy metal atom (Ag).**

```
PLAT971_ALERT_2_C Check Calcd Resid. Dens.  1.44Ang From N00D           1.98 eA-3
```

**Author Response: This Alert is due to presence of residual density in the presence of heavy metal atom (Ag).**

PLAT971_ALERT_2_C Check Calcd Resid. Dens. 0.83Ang From C01I 1.92 eA-3

**Author Response: This Alert is due to presence of residual density in the presence of heavy metal atom (Ag).**

PLAT971_ALERT_2_C Check Calcd Resid. Dens. 0.46Ang From C00V 1.88 eA-3

**Author Response: This Alert is due to presence of residual density in the presence of heavy metal atom (Ag).**

PLAT971_ALERT_2_C Check Calcd Resid. Dens. 0.86Ang From Ag01 1.83 eA-3

**Author Response: This Alert is due to presence of residual density in the presence of heavy metal atom (Ag).**

PLAT971_ALERT_2_C Check Calcd Resid. Dens. 1.87Ang From Ag01 1.81 eA-3

**Author Response: This Alert is due to presence of residual density in the presence of heavy metal atom (Ag).**

PLAT971_ALERT_2_C Check Calcd Resid. Dens. 0.62Ang From O00X 1.80 eA-3

**Author Response: This Alert is due to presence of residual density in the presence of heavy metal atom (Ag).**

PLAT971_ALERT_2_C Check Calcd Resid. Dens. 0.31Ang From C00R 1.77 eA-3

**Author Response: This Alert is due to presence of residual density in the presence of heavy metal atom (Ag).**

PLAT971_ALERT_2_C Check Calcd Resid. Dens. 0.61Ang From C01G 1.77 eA-3

**Author Response: This Alert is due to presence of residual density in the presence of heavy metal atom (Ag).**

PLAT971_ALERT_2_C Check Calcd Resid. Dens. 1.86Ang From Ag02 1.75 eA-3

**Author Response: This Alert is due to presence of residual density in the presence of heavy metal atom (Ag).**

PLAT971_ALERT_2_C Check Calcd Resid. Dens. 0.28Ang From C00Y 1.73 eA-3

**Author Response: This Alert is due to presence of residual density in the presence of heavy metal atom (Ag).**

PLAT972_ALERT_2_C Check Calcd Resid. Dens. 0.67Ang From Ag02 -2.32 eA-3

**Author Response: This Alert is due to presence of residual density in the presence of heavy metal atom (Ag).**

PLAT972_ALERT_2_C Check Calcd Resid. Dens. 0.27Ang From C01L -2.27 eA-3

**Author Response: This Alert is due to presence of residual density in the presence of heavy metal atom (Ag).**

PLAT972_ALERT_2_C Check Calcd Resid. Dens. 0.75Ang From Ag01 -2.15 eA-3

**Author Response: This Alert is due to presence of residual density in the presence of heavy metal atom (Ag).**

PLAT972_ALERT_2_C Check Calcd Resid. Dens. 1.46Ang From Ag01 -2.14 eA-3

**Author Response: This Alert is due to presence of residual density in the presence of heavy metal atom (Ag).**

PLAT972_ALERT_2_C Check Calcd Resid. Dens. 0.38Ang From F5 -2.13 eA-3

**Author Response: This Alert is due to presence of residual density in the presence of heavy metal atom (Ag).**

PLAT972_ALERT_2_C Check Calcd Resid. Dens. 0.97Ang From Ag01 -2.06 eA-3

**Author Response: This Alert is due to presence of residual density in the presence of heavy metal atom (Ag).**

PLAT972_ALERT_2_C Check Calcd Resid. Dens. 0.47Ang From C01G -2.05 eA-3

**Author Response: This Alert is due to presence of residual density in the presence of heavy metal atom (Ag).**

PLAT972_ALERT_2_C Check Calcd Resid. Dens. 1.54Ang From Ag02 -2.00 eA-3

**Author Response: This Alert is due to presence of residual density in the presence of heavy metal atom (Ag).**

```
PLAT972_ALERT_2_C Check Calcd Resid. Dens.  1.61Ang From Ag02          -2.00 eA-3
```

**Author Response: This Alert is due to presence of residual density in the presence of heavy metal atom (Ag).**

```
PLAT972_ALERT_2_C Check Calcd Resid. Dens.  1.31Ang From N00D          -1.91 eA-3
```

**Author Response: This Alert is due to presence of residual density in the presence of heavy metal atom (Ag).**

```
PLAT972_ALERT_2_C Check Calcd Resid. Dens.  0.85Ang From Ag01          -1.85 eA-3
```

**Author Response: This Alert is due to presence of residual density in the presence of heavy metal atom (Ag).**

```
PLAT972_ALERT_2_C Check Calcd Resid. Dens.  0.78Ang From Ag01          -1.84 eA-3
```

**Author Response: This Alert is due to presence of residual density in the presence of heavy metal atom (Ag).**

```
PLAT972_ALERT_2_C Check Calcd Resid. Dens.  1.70Ang From F00F          -1.78 eA-3
```

**Author Response: This Alert is due to presence of residual density in the presence of heavy metal atom (Ag).**

```
PLAT972_ALERT_2_C Check Calcd Resid. Dens.  1.17Ang From C00V          -1.76 eA-3
```

**Author Response: This Alert is due to presence of residual density in the presence of heavy metal atom (Ag).**

```
PLAT972_ALERT_2_C Check Calcd Resid. Dens.  0.77Ang From Ag02          -1.75 eA-3
```

**Author Response: This Alert is due to presence of residual density in the presence of heavy metal atom (Ag).**

```
PLAT972_ALERT_2_C Check Calcd Resid. Dens.  1.43Ang From C012          -1.74 eA-3
```

**Author Response: This Alert is due to presence of residual density in the presence of heavy metal atom (Ag).**

```
PLAT972_ALERT_2_C Check Calcd Resid. Dens.  1.69Ang From C012          -1.74 eA-3
```

**Author Response: This Alert is due to presence of residual density in the presence of heavy metal atom (Ag).**

PLAT972_ALERT_2_C Check Calcd Resid. Dens. 0.67Ang From Ag02 -1.72 eA-3

**Author Response: This Alert is due to presence of residual density in the presence of heavy metal atom (Ag).**

PLAT972_ALERT_2_C Check Calcd Resid. Dens. 1.53Ang From Ag02 -1.71 eA-3

**Author Response: This Alert is due to presence of residual density in the presence of heavy metal atom (Ag).**

PLAT972_ALERT_2_C Check Calcd Resid. Dens. 1.40Ang From Ag02 -1.71 eA-3

**Author Response: This Alert is due to presence of residual density in the presence of heavy metal atom (Ag).**

PLAT972_ALERT_2_C Check Calcd Resid. Dens. 1.58Ang From O00A -1.66 eA-3

**Author Response: This Alert is due to presence of residual density in the presence of heavy metal atom (Ag).**

PLAT972_ALERT_2_C Check Calcd Resid. Dens. 1.48Ang From O00A -1.63 eA-3

**Author Response: This Alert is due to presence of residual density in the presence of heavy metal atom (Ag).**

PLAT972_ALERT_2_C Check Calcd Resid. Dens. 0.30Ang From C00R -1.59 eA-3

**Author Response: This Alert is due to presence of residual density in the presence of heavy metal atom (Ag).**

PLAT972_ALERT_2_C Check Calcd Resid. Dens. 1.72Ang From F005 -1.58 eA-3

**Author Response: This Alert is due to presence of residual density in the presence of heavy metal atom (Ag).**

PLAT977_ALERT_2_C Check Negative Difference Density on H01A . -0.53 eA-3
PLAT977_ALERT_2_C Check Negative Difference Density on H01B . -1.37 eA-3
PLAT977_ALERT_2_C Check Negative Difference Density on H01D . -0.80 eA-3
PLAT977_ALERT_2_C Check Negative Difference Density on H01G . -0.47 eA-3

---

### Alert level G

```
PLAT002_ALERT_2_G Number of Distance or Angle Restraints on AtSite          2 Note
PLAT003_ALERT_2_G Number of Uiso or U(i,j)  Restrained non-H-Atoms         42 Report
PLAT042_ALERT_1_G Calc. and Reported MoietyFormula Strings  Differ     Please Check
              Calc: C26 H15 Ag3 F18 N6 O2
              Rep.: C15 H3 Ag3 F18 N6, C11 H12 O2
PLAT083_ALERT_2_G SHELXL Second Parameter in WGHT  Unusually Large     371.13 Why ?
PLAT172_ALERT_4_G The CIF-Embedded .res File Contains DFIX Records          1 Report
PLAT177_ALERT_4_G The CIF-Embedded .res File Contains DELU Records          6 Report
PLAT178_ALERT_4_G The CIF-Embedded .res File Contains SIMU Records          3 Report
PLAT186_ALERT_4_G The CIF-Embedded .res File Contains ISOR Records          9 Report
PLAT188_ALERT_3_G A Non-default SIMU Restraint Value has been used     0.0100 Report
PLAT188_ALERT_3_G A Non-default SIMU Restraint Value has been used     0.0200 Report
PLAT242_ALERT_2_G Low    'MainMol' Ueq as Compared to Neighbors of       C00W Check
PLAT242_ALERT_2_G Low    'MainMol' Ueq as Compared to Neighbors of       C010 Check
PLAT242_ALERT_2_G Low    'MainMol' Ueq as Compared to Neighbors of       C016 Check
PLAT242_ALERT_2_G Low    'MainMol' Ueq as Compared to Neighbors of       C017 Check
PLAT242_ALERT_2_G Low    'MainMol' Ueq as Compared to Neighbors of       C01A Check
PLAT242_ALERT_2_G Low    'MainMol' Ueq as Compared to Neighbors of       C01I Check
PLAT299_ALERT_4_G Atom Site Occupancy Constrained at ............         0.5 Check
              F2      F6
PLAT301_ALERT_3_G Main Residue  Disorder .............(Resd    1)          2% Note
PLAT432_ALERT_2_G Short Inter X...Y Contact  F6       ..C01L     .       2.68 Ang.
                                         2-x,-1/2+y,3/2-z  =      2_746 Check
PLAT434_ALERT_2_G Short Inter HL..HL Contact F008     ..F00L     .       2.72 Ang.
                                            x,3/2-y,1/2+z  =      4_576 Check
PLAT605_ALERT_4_G Largest Solvent Accessible VOID in the Structure         15 A**3
PLAT720_ALERT_4_G Number of Unusual/Non-Standard Labels ..........         67 Note
              Ag01    Ag02    Ag03    F004    F005    F006    F007    F008
              F009    O00A    N00B    C018    C01E    H01E    C01G    N00M
              F00C    F00F    F00G    F00H    F00J    F00K    F00L    F00N
              N00O    N00I    C00Y    C00S    H00S    C013    F00P    C00Q
              C00R    N00E    N00D    C00V    C00T    H00T    C00U    C00W
              O00X    C00Z    C010    C011    H011    C012    H01A    H01B
              C014    H01C    H01D    C016    C017    C019    H019    C01A
              C01B    H01F    C01C    C01D    H01G    H01H    C01I    C01L
              H01I    H01J    H01K
PLAT860_ALERT_3_G Number of Least-Squares Restraints ............         583 Note
PLAT868_ALERT_4_G ALERTS Due to the Use of _smtbx_masks Suppressed          ! Info
PLAT883_ALERT_1_G Absent Datum for _atom_sites_solution_primary ..     Please Do !
PLAT910_ALERT_3_G Missing FCF Reflection(s) Below Theta(Min)[Deg]=       4.01 Note
                1  0  0,
PLAT912_ALERT_4_G Missing # of FCF Reflections Above STh/L=  0.600        300 Note
PLAT941_ALERT_3_G Average HKL Measurement Multiplicity ..........         2.9 Low
PLAT969_ALERT_5_G The 'Henn et al.' R-Factor-gap value ..........       7.407 Note
              Predicted wR2: Based on SigI**2  4.00 or SHELX Weight 23.85
PLAT978_ALERT_2_G Number C-C Bonds with Positive Residual Density.          0 Info
```

---

   4 **ALERT level A** = Most likely a serious problem - resolve or explain
   5 **ALERT level B** = A potentially serious problem, consider carefully
  61 **ALERT level C** = Check. Ensure it is not caused by an omission or oversight
  30 **ALERT level G** = General information/check it is not something unexpected

   3 ALERT type 1 CIF construction/syntax error, inconsistent or missing data

```
  73 ALERT type 2 Indicator that the structure model may be wrong or deficient
  13 ALERT type 3 Indicator that the structure quality may be low
  10 ALERT type 4 Improvement, methodology, query or suggestion
   1 ALERT type 5 Informative message, check
```

---

It is advisable to attempt to resolve as many as possible of the alerts in all categories. Often the minor alerts point to easily fixed oversights, errors and omissions in your CIF or refinement strategy, so attention to these fine details can be worthwhile. It is up to the individual to critically assess their own results and, if necessary, seek expert advice.

### Validation response form

Please find below a validation response form (VRF) that can be filled in and pasted into your CIF.

```
# start Validation Reply Form
_vrf_DIFMX02_lcz027
;
PROBLEM: The maximum difference density is > 0.1*ZMAX*0.75
RESPONSE: ...
;
_vrf_PLAT082_lcz027
;
PROBLEM: High R1 Value ..................................       0.12 Report
RESPONSE: ...
;
_vrf_PLAT084_lcz027
;
PROBLEM: High wR2 Value (i.e. > 0.25) ...................       0.30 Report
RESPONSE: ...
;
_vrf_PLAT094_lcz027
;
PROBLEM: Ratio of Maximum / Minimum Residual Density ....       2.04 Report
RESPONSE: ...
;
_vrf_PLAT097_lcz027
;
PROBLEM: Large Reported Max.  (Positive) Residual Density       4.60 eA-3
RESPONSE: ...
;
_vrf_PLAT213_lcz027
;
PROBLEM: Atom F00K            has ADP max/min Ratio .....        3.2 prolat
RESPONSE: ...
;
_vrf_PLAT220_lcz027
;
PROBLEM: NonSolvent   Resd 1  F   Ueq(max)/Ueq(min) Range        3.1 Ratio
RESPONSE: ...
;
_vrf_PLAT234_lcz027
;
PROBLEM: Large Hirshfeld Difference F00N     --C010     .       0.17 Ang.
```

```
RESPONSE: ...
;
_vrf_PLAT362_lcz027
;
PROBLEM: Short  C(sp3)-C(sp2) Bond  C018     - C00W     .       1.40 Ang.
RESPONSE: ...
;
_vrf_PLAT906_lcz027
;
PROBLEM: Large K Value in the Analysis of Variance ......     14.550 Check
RESPONSE: ...
;
_vrf_PLAT911_lcz027
;
PROBLEM: Missing FCF Refl Between Thmin & STh/L=    0.600         79 Report
RESPONSE: ...
;
_vrf_PLAT934_lcz027
;
PROBLEM: Number of (Iobs-Icalc)/Sigma(W) > 10 Outliers ..          1 Check
RESPONSE: ...
;
_vrf_PLAT977_lcz027
;
PROBLEM: Check Negative Difference Density on H01A      .      -0.53 eA-3
RESPONSE: ...
;
# end Validation Reply Form
```

---

**PLATON version of 17/09/2025; check.def file version of 17/09/2025**

---

# duplicate check

**No duplication found**

---

**Datablock lcz027** - ellipsoid plot

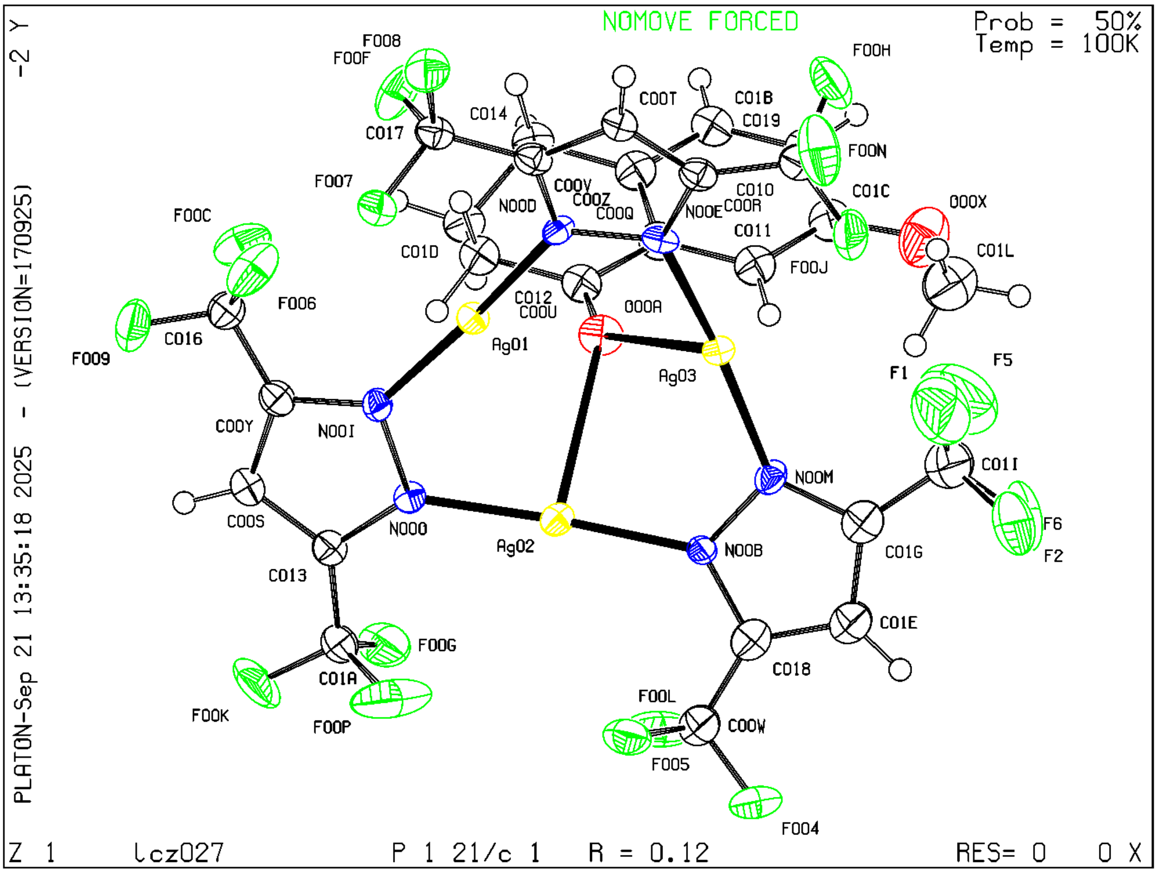

Supplement: Supplementary file 2 — Supporting File 2: anie72462‐sup‐0002‐DataFile.zip. [file ANIE-65-e1218503-s002.zip › supplementary_materials_crystallographic_data submit.zip!/cif_and_checkcif(1-114)/Ag3Pz3_27/Ag3Pz3_27_checkcif.pdf]
